# Sensor-based systems for early detection of dementia (SENDA): a study protocol for a prospective cohort sequential study

**DOI:** 10.1186/s12883-020-01666-8

**Published:** 2020-03-07

**Authors:** Katrin Müller, Stephanie Fröhlich, Andresa M. C. Germano, Jyothsna Kondragunta, Maria Fernanda del Carmen Agoitia Hurtado, Julian Rudisch, Daniel Schmidt, Gangolf Hirtz, Peter Stollmann, Claudia Voelcker-Rehage

**Affiliations:** 1 Department of Sports Psychology (with focus on prevention and rehabilitation), Institute of Human Movement Science and Health, Faculty of Behavioural and Social Sciences, Chemnitz University of Technology, Thüringer Weg 11, 09126 Chemnitz, Germany; 2 Department of Human Locomotion, Institute of Human Movement Science and Health, Faculty of Behavioural and Social Sciences, Chemnitz University of Technology, Chemnitz, Germany; 3 Department of Digital Signal Processing and Circuit Technology, Faculty of Electrical Engineering and Information Technology, Chemnitz University of Technology, Chemnitz, Germany; 4 Department of Analysis, Faculty of Mathematics, Chemnitz University of Technology, Chemnitz, Germany; 5 Department of Neuromotor Behavior and Exercise, University of Münster, Münster, Germany

**Keywords:** Dementia, MCI, Early detection, Motor performance, Gait analyses, Sensitivity, Proprioception, EEG

## Abstract

**Background:**

Dementia and cognitive decline are serious social and economic burdens. An increase in the population of older people, as well as longer lifespans mean that numbers of dementia cases are exponentially rising. Neuropathological changes associated with dementia are thought to appear before the clinical manifestation of cognitive symptoms, i.e., memory impairments. Further, some older adults (OA) experience cognitive decline before it can be objectively diagnosed. For optimal care of these patients, it is necessary to detect cognitive decline and dementia at an early stage. In this vein, motor, sensory, and neurophysiological declines could be promising factors if found to be present before the onset of cognitive impairment. Hence, the objective of the SENDA study is to develop a multi-dimensional sensor-based instrument that allows early detection of cognitive decline or dementia in OA with the help of cognitive, sensory, motor, and neurophysiological parameters before its clinical manifestation.

**Methods/design:**

In the cohort sequential study, participants are assigned to one of three study groups depending on their cognitive status: 1. cognitively healthy individuals (CHI), 2. subjectively cognitively impaired persons (SCI), or 3. (possible) mildly cognitively impaired persons (pMCI, MCI). All groups take part in the same cognitive (e.g., executive function tests), motor (e.g., gait analyses, balance tests), sensory (e.g., vibration perception threshold test, proprioception tests), and neurophysiological (e.g., electroencephalograms) measurements. Depending on the time at which participants are included into the study, all measurements are repeated up to four times in intervals of 8 months within 3 years to identify associations with cognitive changes over time.

**Discussion:**

This study aims to detect possible motor, sensory, neurophysiological, and cognitive predictors to develop an early screening tool for dementia and its pre-stages in OA. Thus, affected persons could receive optimal health care at an earlier time point to maintain their health resources.

**Trial status:**

The study is ongoing. The recruitment of participants will be continued until May 2020.

## Background

Dementia is a common age-related neurodegenerative disease whose prevalence is increasing globally. According to the German Alzheimer Society e.V. [1], the number of dementia cases in Germany will have risen to three million by 2050. In addition to the personal cost, the disease causes substantial economic and social burdens [2]. Early diagnosis of dementia and its pre-stages could alleviate these by enabling sustainable disease management and optimal health care for affected individuals.

Although no effective treatment of dementia exists yet, early diagnosis has been shown to enable interventions which slow down disease progression (i.e. physical activity interventions [3] or pharmaceutical interventions [4]). Early diagnosis provides the opportunity to start treatment before neurodegeneration has progressed and with only minimal disease pathology present [5]. The deterioration of cognitive functions, e. g., memory, attention, or executive functions, is a typical symptom of this illness [6]. Additionally, patients with dementia show anomalies in their social behavior and activities of daily living (ADL) [6]. Mild cognitive impairment (MCI) is classified on a continuum between cognitive changes of normal aging and symptoms of dementia [7]. In this vein, people with MCI have a 10-fold increased risk of developing dementia [8]. Patients with MCI are characterized by the following criteria: (1) concerns about changes in cognition by themselves or someone else, (2) impairments in at least one cognitive domain, and (3) no problems in ADL [9]. Cognitive impairments most often pertain to memory, but can also include other cognitive domains, such as executive functions, attentional control, language skills, or visuospatial skills [9]. Interestingly, a substantial amount of older people report memory loss and other cognitive deficits even in the absence of objective cognitive impairments [10]. The condition has been classed as SCI, meaning subjective cognitive impairment, as people subjectively experience worsening of their cognitive performance compared to prior performance levels while they still perform within normal range on standard clinical assessments of cognition [11]. SCI has been shown to triple the risk of Alzheimer’s disease [12], is associated with underlying dementia neuropathology [13], and as such could be considered an even earlier pre-clinical stage of dementia [11].

Brain imaging, e.g., computed tomography or magnetic resonance imaging, laboratory tests, and cognitive or neuropsychological tests are standard methods of diagnosing dementia [6, 14]. However, by the time individuals receive the diagnosis, cognitive impairments will generally have progressed [5, 15]. Neuropathological changes associated with dementia have been found to develop before the clinical manifestation of cognitive symptoms, i.e., memory impairments [5], and might be expressed in SCI or even MCI. Since the costs are too high to use neuroanatomical and biological markers for diagnosis [9], it is worthwhile to explore whether behavioral markers, other than cognitive performance, can be used to successfully predict the development of dementia. If successful, it would provide a low cost and easy to apply approach to screening for dementia at the pre-clinical stage, and enable appropriate interventions to be established to delay its clinical manifestations. Consequently, current research aims to determine prodromal markers for early detection of dementia, for example, changes in the motor (e.g. abnormalities in gait) or the sensory systems [16].

With regard to motor control, persons with MCI present a transitional stage between healthy controls and patients with early Alzheimer’s disease [17]. For example, identifying abnormalities in gait parameters are a key focus in early screening for dementia [18–20]. A meta-analysis of Bahureska et al. [18] revealed that lower gait velocity, termed senile gait [21, 22], seems to be a marker to discriminate between MCI and healthy controls [19]. Additionally, poor performance walking under more complex conditions, such as dual-task conditions, has been associated with higher risk of developing dementia [19, 23, 24].

Furthermore, other motor changes might be used for predicting dementia, e.g., dynamic balance control, finger dexterity, and cutaneous sensitivity*.* Many anatomical structures (e.g., the brainstem, spinal cord, or the primary somatosensory cortex [25–29]), associated with processing cutaneous sensations, are negatively affected in dementia, early dementia, or its precursor MCI. To date, however, there are only few studies which investigate cutaneous sensitivity in MCI patients or dementia diseases [30]. Cutaneous sensitivity is essential for motor performance [31], gait [32], and balance [33]. Quasi-static balance [34] has already been identified as a prodromal marker of dementia [18], whereas dynamic balance with unexpected perturbations has not yet been explored in patients with MCI. Changes in finger dexterity could be predictors for the development of dementia [35], independently from age-related changes [36]. For instance, Rabinowitz and Larner [37] revealed that patients with MCI or dementia show an increase in duration and variability of the finger-touch phase during finger tapping compared to cognitively healthy OA.

Furthermore, neurophysiological techniques, including electroencephalography (EEG), enable detection of functional changes in brain activity at an early stage of dementia [38, 39]. Resting state EEG reveals differences between persons with dementia or pre-clinical dementia and healthy OA [38, 39]. The limited number of longitudinal studies have identified the mean frequency of the total spectrum [40], relative beta power [41], relative alpha power [41–44], relative theta power [40, 42, 45], coherence across all frequencies [40], and coherence in the delta band [46] as possible predictors of cognitive decline. Unfortunately, there is not yet a clear consensus about which parameters best predict dementia or how to translate these findings into cut-offs for individual diagnosis.

In conclusion, early detection of dementia at the presymptomatic stage of disease using prodromal markers is important to detect progressive changes of the central nervous system and to initiate targeted and optimal health care as early as possible. There are, however, only few studies which investigate different markers (e.g., biomarkers, cognitive markers) to detect cognitive decline or the transition from MCI to dementia [47–51]. Gomar et al. [47] examined different biomarkers (e.g., total tau, Aβ1–42), cognitive markers (working memory), and risk factors (APOE genotype) in one study to predict transition from MCI to Alzheimer’s disease. They were able to show that cognitive markers predict these transitions more than most biomarkers. This was also shown in a 4 year follow-up data phase [48]. Another longitudinal study (The Sydney Memory and Ageing Study) by Lipnicki et al. [52] revealed that older age, slower walking speed, and APOE ε4 carrier at baseline were associated with MCI or dementia after 6 years. In a current gait and balance platform study (part of the Ontario Neurodegenerative Research Initiative (ONDRI) by Montero-Odasso et al. [53]), motor-cognitive profiles across neurodegenerative diseases, e. g. Alzheimer’s diseases or MCI, will be identified over 3 years using gait and balance tests. However, to our knowledge, other than the ONDRI study [54], there are no other studies investigating cognitive, motor, sensory, and neurophysiological markers in combination to develop a multi-dimensional instrument to predict cognitive decline or dementia. Therefore, the objective of the current study is to develop such a multi-dimensional sensor-based instrument to detect cognitive decline or dementia in older adults with the help of several cognitive, sensory, motor, and electroencephalogical parameters in a longitudinal cohort. The results of this study will lead to a better understanding of the different prodromal markers and their interaction, and might help to predict MCI or dementia.

This study is named “Sensor-based systems for early detection of dementia (SENDA)” and is funded by the European Social Fund and the Sächsische AufbauBank-Förderbank (SAB) of the Free State of Saxony (Project-Number: 100310502).

## Methods

### Study aims

The following main research question investigated in this study is:

Which cognitive, sensory, motor, and neurophysiological variables are predictors of the transition from subjective cognitive impairment or MCI to dementia in comparison to age-matched healthy OA?

The objective of the SENDA study is to develop a multi-dimensional sensor-based instrument based on the stated variables or their combination to detect cognitive decline or dementia in OA.

### Participants and procedures

Participants were recruited via local newspaper articles and the website of the Chemnitz University of Technology. In addition, we received 1500 names and addresses of men and women aged ≥ 80 years from the registration office of the city of Chemnitz to enable initial contact for potential study participation. A study hotline was set up for anyone interested in study participation to call. Trained project staff determine eligibility for study participation in telephone interviews following the inclusion and exclusion criteria outlined below. People who fulfill the inclusion criteria are invited to participate in the study by mail.

#### Inclusion and exclusion criteria

Men and women aged ≥ 80 years and with their principal residence in the city of Chemnitz and surrounding areas are included in the study. Participants must be able to visit the lab independently or with the help of an accompanying person. They must be able to walk by themselves, but the use of a walking aid is allowed. Further criteria for inclusion in the study are basic knowledge of German and passing hearing and vision screening tests. Participants are excluded from the study if they present any of the criteria listed in Table 1.

**Table 1:** Exclusion criteria of the study

**Exclusion criteria**	
- Medically prohibited to be physically active - Diagnosed psychological disorders, such as major depression, or neurocognitive disorders, such as dementia (MoCA-score < 19) - Permanent impairments due to a stroke or brain surgery - Other neurological diseases, such as epilepsy, Parkinson, or neuropathy - Severe diseases of the cardiovascular system (e.g., cardiac arrhythmia, arterial occlusive disease, heart failure) - Severe diseases of the respiratory system (e.g., COPD stage 4, severe asthma) - Severe diseases of the musculoskeletal system (e.g., arthritis, orthopedic operations in the last 6 months) - Diabetes with diagnosed neuropathy - Substance abuse - Difficulties understanding language or speech - Participant of other clinical studies, e.g., for clinical testing of new anti-dementia drugs	

#### Study design

**Fig. 1:**
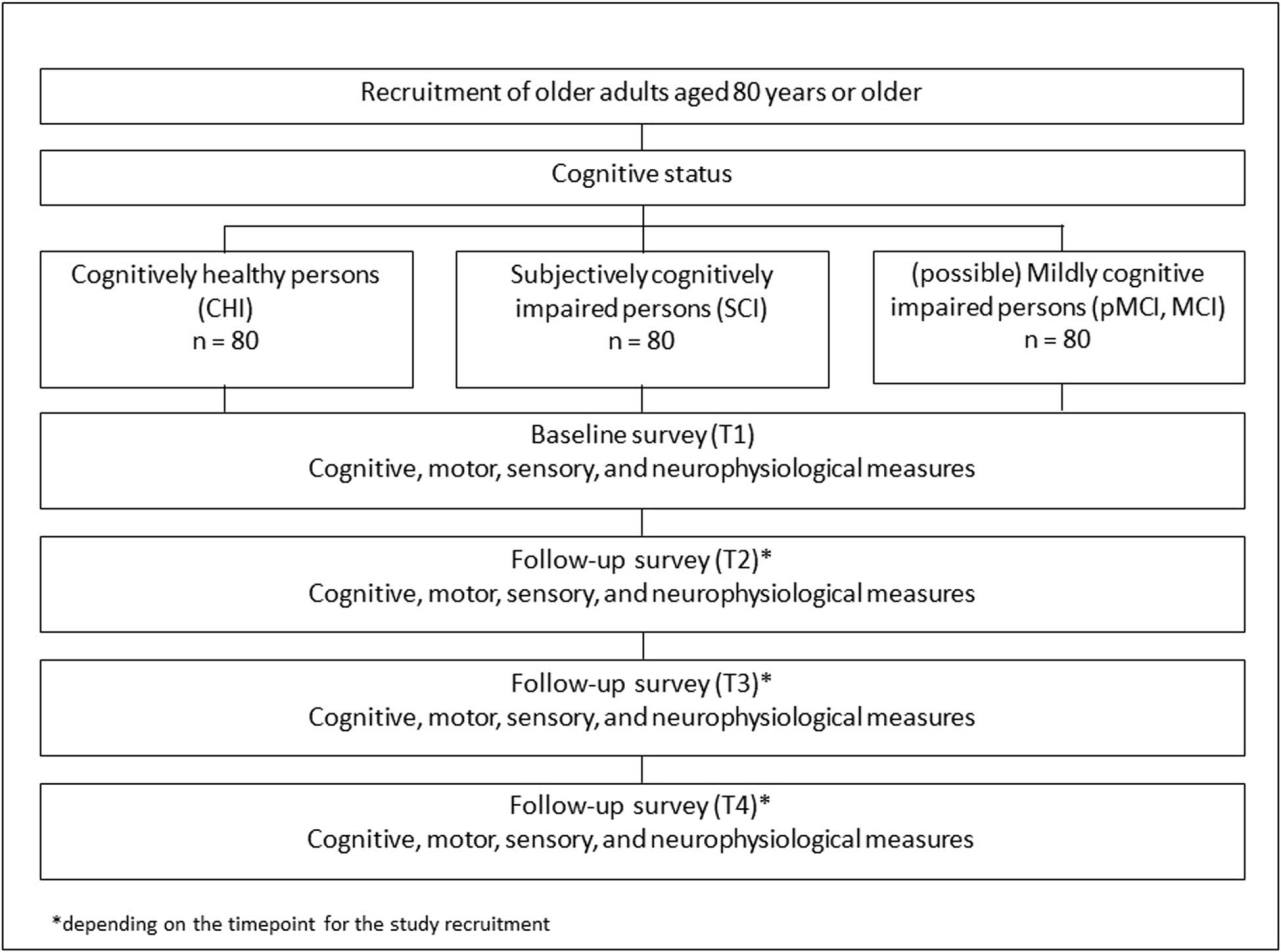
Study design

The SENDA study is designed as a prospective cohort sequential study. After successful screening for study eligibility, participants are assigned to one of three study groups depending on their cognitive status based on their MoCA (Montreal Cognitive Assessment), CERAD-Plus (Consortium to Establish a Registry for Alzheimer’s Disease), and the FLei (‘Fragebogen zur geistigen Leistungsfähigkeit’, questionnaire for complaints of subjective cognitive disturbances) scores: 1. cognitively healthy individuals (CHI), 2. subjectively cognitively impaired persons (SCI), 3. possible mildly cognitively impaired persons due to inconclusive test results (pMCI), or mildly cognitively impaired persons (MCI). During the study period participants of four cohorts will be recruited at different time points (see Table 2). All participants complete the same cognitive, motor, sensory, and neurophysiological tests. Depending on the time point of study entry, all tests are repeated up to four times (time points: T1, T2, T3, T4) in intervals of 8 months within 3 years to identify associations with cognitive changes over time (see Fig. 1 for the study design and Table 2). The interval of 8 months was chosen according to Chamberlain et al. [55]. Only this frequency of follow-ups enables the measurements to be repeated up to three times in the defined funding period of 3 years. One test consists of three examination days of 1 to 2 h each.

**Table 2:** Time points of study recruitment of the four cohorts and number of follow-up surveys (T1: Baseline; T2-T4: Follow-up surveys; ‘X’: participation; ‘-’: no participation)

Cohorts	T1	T2	T3	T4
Cohort 1 (begin: February 2018)	X	X	X	X
Cohort 2 (begin: July 2018)	X	X	X	-
Cohort 3 (begin: January 2019)	X	X	-	-
Cohort 4 (begin: January 2020)	X	-	-	-

### Outcome measures

Participants are invited to the labs of the study center in Chemnitz to complete the baseline (T1) and follow-up measurements (T2, T3, T4) as shown in Fig. 1. At all time points, participants undergo proven and standardized assessments, including different motor, sensory, cognitive, and electroencephalogical assessments.

### Cognitive assessments

#### Montreal Cognitive Assessment/ Consortium to Establish a Registry for Alzheimer’s Disease

To identify cognitive decline, we assess global cognition using the MoCA (Montreal Cognitive Assessment [56]) and the CERAD-Plus (Consortium to Establish a Registry for Alzheimer’s Disease [57]) tools. MoCA is a short screening tool for measuring mild cognitive impairment, i.e., in memory, attention, or executive functions. Participants can reach a maximum of 30 points [58]. The cut-off between healthy and mild cognitive impairment is set at 26 in accordance with the recommendations from Nasreddine et al. [56], which means individuals with a score of 25 or lower are considered impaired.

CERAD-Plus is a reliable and valid assessment of Alzheimer’s disease and consists of different neuropsychological tests, such as the Mini-Mental State Examination, verbal fluency, Boston Naming, word list learning, recall and recognition, constructional praxis and recall, and trail making tests A and B [57]. To detect objective cognitive impairments according to CERAD-Plus, we will compare scores in each subtest (excluding MMSE) to the age, education, and gender-controlled reference norms. Following recommendations of the National Institute on Aging-Alzheimer’s Association workgroups on diagnostic guidelines for Alzheimer’s disease [9], performances worse than 1.5 SD below the norm in one or more subtests are considered objective cognitive impairments.

#### Questionnaire for subjective assessment of mental performance (FLei)

We use the FLei (‘Fragebogen zur geistigen Leistungsfähigkeit’ [59]) to assess subjective cognitive status. The questionnaire employs 30 questions about cognitive challenges in everyday life, including items about executive functions, memory, attention, and 5 control items. Participants are asked to indicate the frequency of these challenges on a scale from 0 (‘never’) to 5 (‘very often’) and a sum score is then calculated (range 0–120). Participants with a score of 31 or higher and no objective cognitive impairment are categorized as only subjectively impaired in their cognition. This cut-off was chosen because 30 points are reached when a participant chooses 1 (‘seldom’) for every item. This is in accordance with data in other studies showing that participants on average score at the lower end of the answer range (for example *M* = 36.3 for OA with objective impairment [60] and *M* = 28.2 for a general population representative sample [61]). After completing data collection, we will also use a data driven approach to determine a suitable cut-off for our sample and, if needed, adjust the assessment of subjective cognitive status.

#### Cognitive status

All prior introduced neuropsychological assessments are used to establish the cognitive status of each participant at baseline and each follow-up. All criteria including cut-offs can be found in Table 3. Cognitive decline is defined as a change from CHI to SCI, pMCI, or MCI, as well as a change from SCI to pMCI or MCI or from pMCI and MCI to dementia. Individuals are categorized as having dementia when they receive a clinical dementia diagnosis outside of the study at any follow-up. Furthermore, participants are categorized as having dementia if they score less than 19 points in the MoCA [62] and perform worse than 1.5 SD below the norm in multiple cognitive domains of the CERAD-Plus. If so participants are advised to visit their general practitioner for further evaluation regarding dementia and to give a feedback to the study coordinator of SENDA as soon as possible. Additionally, functional limitations in ADL and the presence of depression will also be taken into account (see section ‘Questionnaire battery’).

**Table 3:** Criteria for cognitive status of the participants

Group	Cognitive Status	Measures		
		MoCA	CERAD-Plus	Flei
Group 1	CHI	30–26	All tests within normal range (≤ 1.5 SD)	≤ 30
Group 2	SCI	30–26	All tests within normal range (≤ 1.5 SD)	> 30
Group 3	pMCI	30–26	1 or more tests below normal range (≥ 1.5 SD)	not considered
		< 26	All tests within normal range (≤ 1.5 SD)	not considered
	MCI	< 26	1 or more tests below normal range (≥ 1.5 SD)	not considered

#### Digit Symbol Substitution Test

The Digit Symbol Substitution Test (DSST), as a part of the Wechsler Adult Intelligence Scale, is a neuropsychological test for response speed, sustained attention, visual spatial skills, and set shifting. The pencil paper task is timed at 90 s. Participants have to write down the correct symbol which is paired to a series of digits from 1 to 9. The correct number-symbol matches are then calculated [63].

#### Flanker task

We use a modification of the Eriksen flanker task [64] to study attentional control and response inhibition, two executive function skills known to be impaired in patients with MCI and dementia [65, 66]. Stimuli consist of a center disk surrounded by four flanker disks set against a black background. Participants are asked to ignore the flanker disks (blue, red, or green) and react only to the center disk (red or green) by pressing the button of the correct color. The task consists of three blocks of 100 trials. One trial consists of a fixation cross (300 ms), a blank screen (200 ms), stimulus presentation (200 ms), a blank screen during the response interval (terminated by button press, maximal 3000 ms), and a blank screen (randomly chosen between 500 to 800 ms). Outcomes include response times of correct trials and accuracy.

With respect to cognitive testing, standardized methods with known psychometric properties, as well as data available about practice effects were chosen. All have good to excellent test-retest-reliability (r = .92 for MoCA [56], r = .88 for DSST [67], r = .53–.91 for subtests of the CERAD-Plus [68] and ICC = .61–.74 for RT of different trial types in the Flanker task [69]). In addition, practice effects are usually smaller for longer intervals and older participants [70].

#### Single and dual-task cognitive performance

All participants perform the modified Serial Sevens Test (SST) and Verbal Fluency Test (VFT) during single-task (while seated for 15 s) and dual-task conditions (during gait), to evaluate the cost of dual-tasking for cognitive functioning. To minimize the effects of learning the order of single and dual tasks were randomized compared to Montero-Odasso et al. [23] and Muir et al. [24]. The number of correct answers is recorded.

The modified SST [71] is a test of cognitive function. During the SST, participants are asked to successively count backwards aloud by increments of 7, starting at either 283 or 213. Due to poor cognitive functioning of most participants, a simpler version of the SST is administered, in which participants have to simultaneously count backwards by increments of 3, starting at either 153 or 183 and by increments of 1, starting at either 200 or 300.

The VFT is an additional test of cognitive function and part of the MoCA [56]. The VFT is a phonemic fluency test, in which participants are asked to generate as many words as possible within a specified time, starting with a specific letter, in this study with ‘K’ or ‘M’. Names or numbers or the same word stem are not allowed. Verbal fluency has been shown to be reduced in elderly persons with mild cognitive impairments as compared to their non-impaired persons [72].

### Neurophysiological measures

#### EEG recording

We record electroencephalograms (EEGs) in all participants with an actiCHamp system (Brain Products GmbH, Gilching, Germany) using 32 electrodes positioned according to the modified 10–20 system (Fp1, Fp2, F7, F3, F4, F8, FC5, FC3, FC1, FC2, FC4, FC6, T7, C3, Cz, C4, T8, CP5, CP3, CP1, CP2, CP4, CP6, P7, P3, Pz, P4, P8, O1, Oz, O2 with reference to Fz and a forehead ground electrode). We keep electrode-skin impedance below 25 kΩ, which is suitable for active electrodes [73]. All data are acquired at 500 Hz sampling rate in continuous recording mode. EEG is recorded during (1) resting with eyes open for 4 min, (2) resting with eyes closed for 2 min, and (3) three fine motor tasks (see below), and (4) a flanker task. Measurements take place in an electrically and acoustically shielded room with lights turned off during rest and dimly lit during task conditions. We monitor participants’ level of consciousness online in real time and annotate changes and artifacts in the EEG protocol. Total recording time is about 60 min and includes individual breaks between tasks.

#### Resting state EEG

For the rest conditions, participants are instructed to sit relaxed on a chair with both hands resting comfortably on the table in front of them. They are asked to first look at a white fixation cross at the center of a black screen for 4 min and to then close their eyes for 2 min. Similar resting state protocols are often used in aging and dementia research [74–77].

### Motor performance

#### Gait analysis

Spatiotemporal gait parameters (i.e. gait velocity, step length, step width) are collected using a walkway system for optical detection (Optogait®, Microgate, Bolzona-Bozen, Italy). Each transmitting and receiving bar consists of 96 LEDs communicating on an infrared (visible) frequency with the same number of LEDs on the opposite bar. The walking distance of each walk is 12 m and includes a turning point after 6 m. Width of the track is 1 m. Participants start 1 m before the beginning of the pathway and stop 1 m past the end. All participants perform the following walking blocks in the same order after one test trial: (1) preferred walking speed (two separate walks), (2) fast walking (one walk); and (3) dual-task walking (preferred walking and cognitive task, four separate walks). Gait performance is assessed by measuring, e.g., gait velocity, step length, and step width, as well as the variability of these parameters. All measured data are recorded and saved for analysis by the Optogait software.

Additionally, we use Kinect and XPCV framework (XPCV-Cross Platform Computer Vision Framework; www.xpcv.de) to record the 3D gait data of the participants for all conditions of the different walks. Acquired data is pre-processed to generate heat maps, and annotation assignment is done. This pre-processed data is used to train our deep learning algorithm to estimate the 3D pose of the person with our 3D pose estimation model, by several different architectures of Convolutional Neural Networks (CNN). Gait parameters are defined and abnormalities are ascertained with factors such as mean stride time, mean stride length, mean stance duration, or mean swing duration.

##### Dual-task walking

The modified SST and VFT are performed during preferred walking speed using the Optogait system. During dual-task walking, participants are instructed to keep walking even if they cannot solve the cognitive task. The selection of the dual-task conditions is based on current research [19, 53].

#### Balance tests

**Fig. 2:**
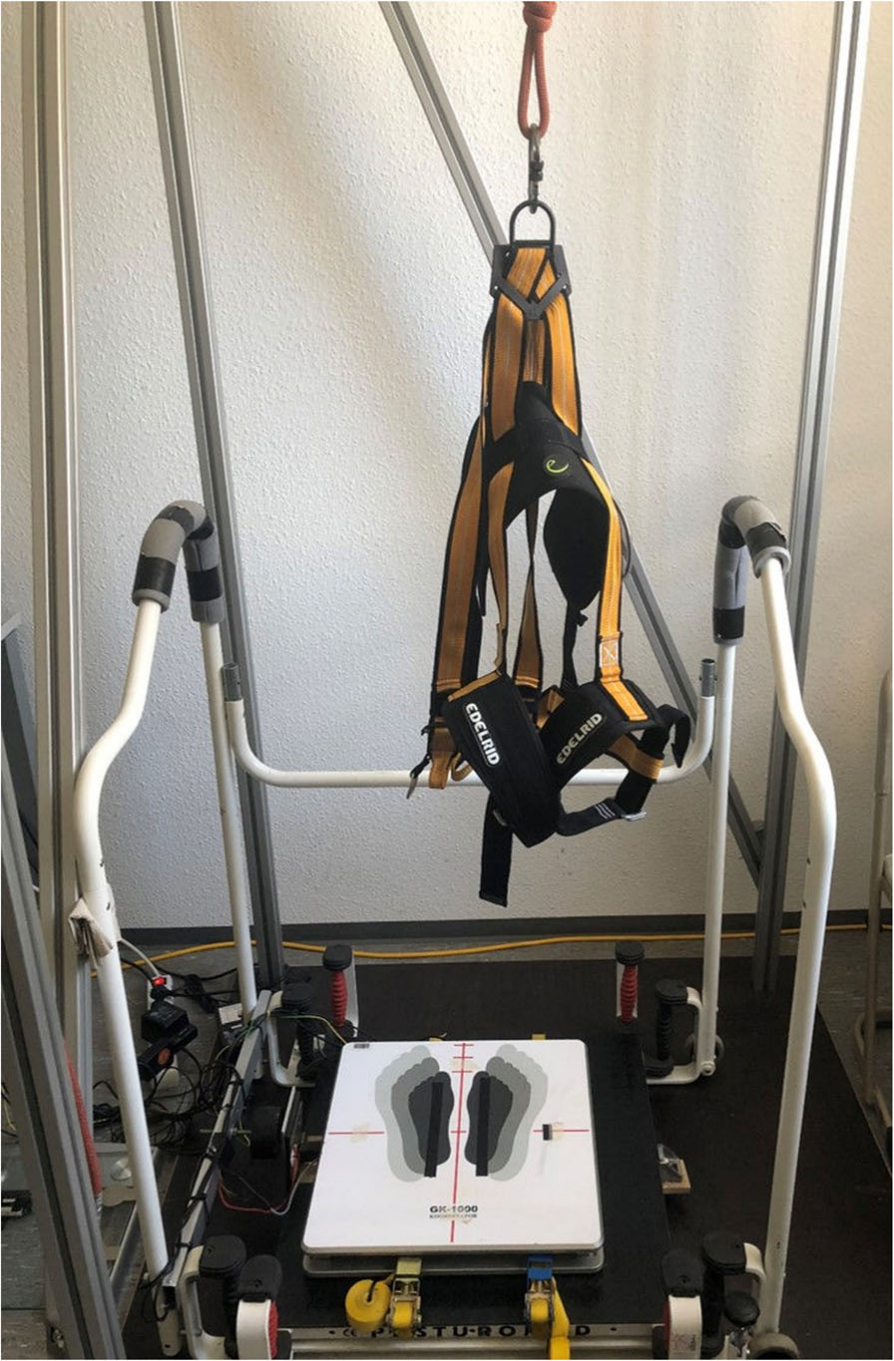
Balance set-up with Posturomed, force-platform, and safety belt

To measure balance tasks, we implement a self-built, customized balance setup test (Fig. 2). The balance setup is made up of a force-platform (IMM Holding GmbH, Germany; 1 kHz), which is installed directly on top of the bottom-platform of a Posturomed device (Haider Bioswing GmbH, Germany). The force-platform is also equipped with heating elements to keep the surface temperature at 25 °C. The bottom-platform of the Posturomed is mobile in the horizontal direction and suspended vertically. To perform quasi-static tests, the bottom-platform is locked in place, so as to prevent movements. To enable unexpected perturbations (dynamic balance tasks), the Posturomed is equipped with an electro-magnet, which holds the bottom-platform in place after shifting it 20 mm out of its neutral position, according to Germano et al. [33]. Unexpected perturbations are induced by manually triggering the electro-magnet, causing the bottom platform to be released and to swing until it again reaches the neutral position. Moreover, the setup also includes a single axis accelerometer ADXL78 (Analog Devices Inc., USA), which is used to detect the reversal points of the oscillating bottom-platform. Participants are secured with a safety belt during all balance tests, which is a built-in safety feature included to prevent falls or other injuries. The balance setup exhibits a good inter- and intra-day reliability [78].

##### Quasi-static balance tests

Participants perform two different balance tasks to measure quasi-static abilities. The first balance task tests participants’ quasi-static balance ability during three conditions: double leg stance (eyes opened and eyes closed) and single leg stance (eyes open). For the double leg stance tests, trials of 25 s are performed and participants are instructed to keep their knees straightened but not locked, and to keep their arms hanging down. They are also asked to evenly distribute their body weight on both feet, keeping them hip width apart. For single leg stance tests, trials of 12 s are collected and participants are asked to stand on their dominant leg while flexing their contra-lateral lower limb backwards and keeping their upper limbs hanging down. To become accustomed to the apparatus, participants perform one practice trial per condition. Then, three trials per condition are collected for data analysis in randomized order.

The second quasi-static balance task is the so-called Limits-of-Stability-Test [79]. Participants are asked to stand as still as possible in their normal posture, with their arms by their sides and eyes opened. After an acoustic signal from the experimenter, they lean forward as far as possible and stay inclined for 10 s. Inclinations are accomplished without lifting toes or heels, and with minimal bending at the hip or knees. Furthermore, the trunk is kept almost straight. One practice trial is performed and another three valid trials are included for data analysis.

##### Dynamic balance tests

The dynamic balance tests investigate the ability to withstand unexpected perturbations in the medio-lateral and anterior-posterior directions [33, 78]. Participants are instructed to look straight ahead while keeping their knees straightened but not locked, and to keep their upper limbs hanging down at both sides, eyes opened. Feet are positioned hip-width apart at the center of the plate. After the experimenter presses a manual trigger, the bottom platform is released, initiating the unexpected perturbation. Subsequently, the bottom platform swings horizontally until it again reaches the neutral position. Participants are asked to maintain or regain their balance while the platform is in motion. The dominant foot is positioned towards the electro-magnet during the tests in the medio-lateral direction. For the anterior-posterior direction, participants stand on the plate with their heels pointing toward the electro-magnet. To become accustomed to the apparatus, each participant performs six trials (three in each direction) before data collection begins. Collecting in a randomized order, the three following valid trials per condition are included in the data analysis.

#### Fine motor tasks

Participants carry out three fine motor tasks: (1) force modulation of a precision grip with thumb and index finger (similar to the set-up of Voelcker-Rehage and Alberts [80]), (2) tapping with the index finger of the dominant hand (based on Rabinowitz and Lavner [38]), and (3) connecting dots on a touchscreen with a touch pen / tracing (as studied by Yan [81]).

**Fig. 3:**
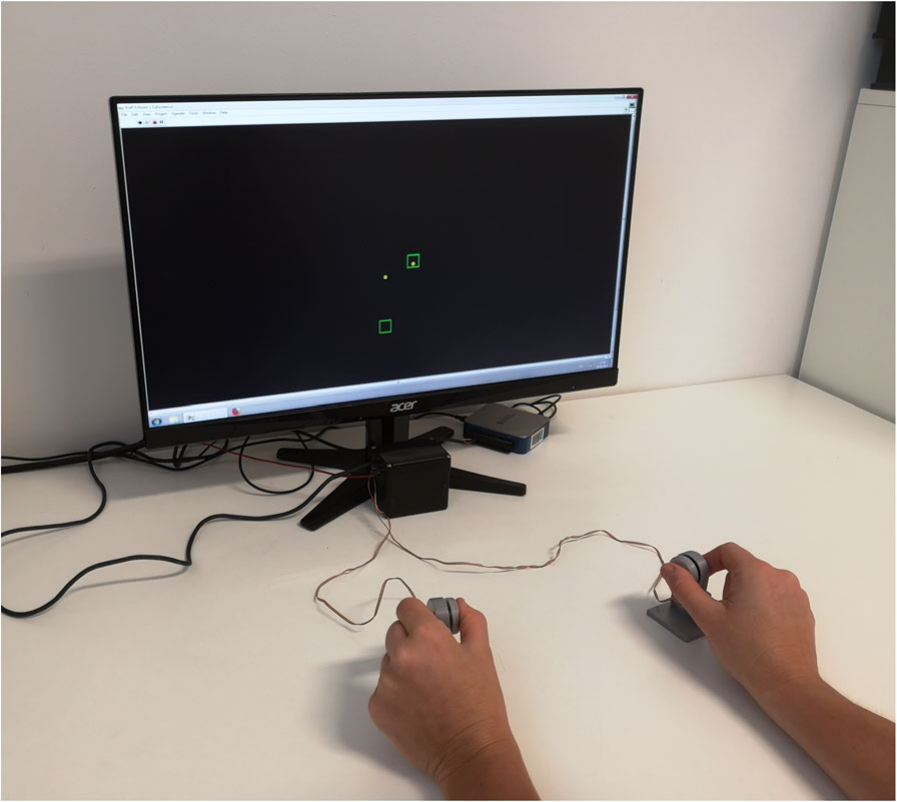
Set-up for fine motor task “force modulation”

To collect data for the fine motor task (1), two compression load cells with a diameter of 29.5 mm, a depth of 8 mm, and a measurement range of 0–22.5 kg (Manufacturer: Measurement Specialties Inc., Hampton, VA, USA; Model: FX-1901-0001-50 L) are used (cf. [82] for comparable unimanual setup). Signals are pre-amplified (using a customized voltage amplifier), digitally converted, and sampled at a frequency of 120 Hz, using a NI-DAQ USB-6002 (National Instruments, Austin, TA, USA). For programming the experimental procedures, i.e., data acquisition and real time visual feedback, a customized LabView 2015 (National Instruments, Austin, TA, USA) script is used. Force transducers are placed on a table in front of the participants, which are seated at a distance of 60 cm in front of a 23.8 in. monitor (hardware resolution 1920 × 1080 pixels). This monitor produces real-time feedback about actual force levels of the participants and target forces that need to be met (see Fig. 3). Feedback about the magnitude of the applied force to both sensors is indicated by two small dots that move up when more force is applied and down when less force is applied. Squares (width and height: 12.5 mm) are displayed on the screen to indicate reference values (see Fig. 3). The scale of the display is adjusted with respect to individual force ranges and the size of the target box corresponds to 0.6% of maximum voluntary contraction (MVC). The aim of the force modulation of a precision grip task is to assimilate the force to a sine wave (ranging from 5 to 12% of MVC of the dominant hand). In our task, the target sine wave is visualized on the screen by the squares which, depending on the condition, are either moving up and down (frequency 0.2 Hz) or are held constant. Participants have to modulate their force to try and keep the dot in the box. This task is performed bimanually and unimanually. The bimanual condition consists of 34 trials (20 s each) overall and includes five conditions: (1) inphase – the target sine waves move simultaneously; (2) antiphase – the target sine waves move inversely, when the right sine wave is on the maximum, the left sine wave is on the minimum; (3) constant – a constant symmetric force with both hands at 12% of MVC (boxes do not move), (4) left hand applies a constant force at 12% of MVC and the right hand follows an alternating sine-wave force pattern between 5 and 12% of MVC, and (5) right hand applies a constant force at 12% of MVC while the left hand follows an alternating sine-wave force pattern between 5 and 12% of MVC.

**Fig. 4:**
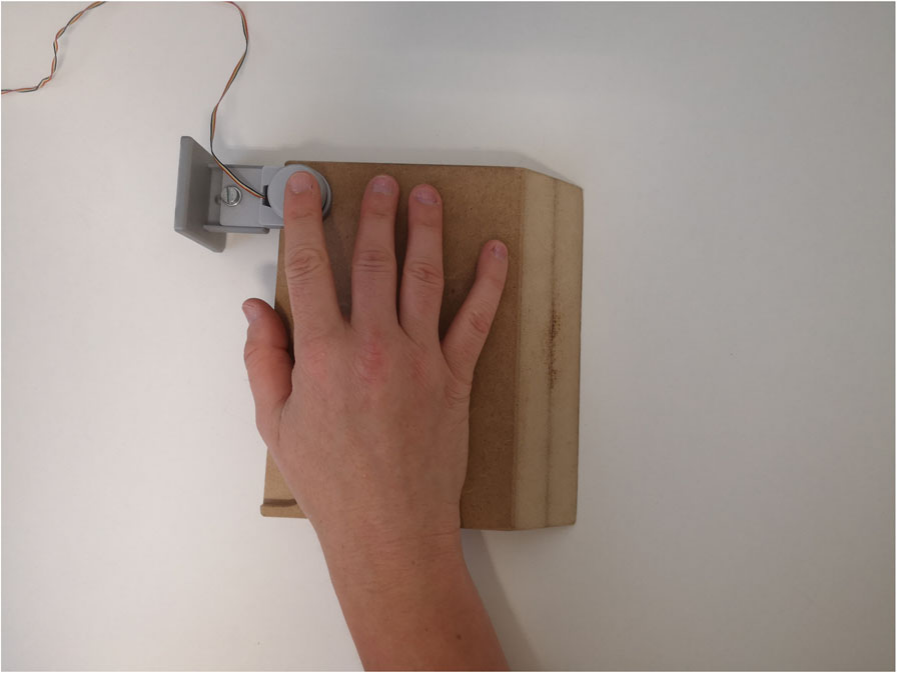
Set-up for fine motor task “finger tapping”

For the fine motor task (2) “finger tapping”, one of the two force transducers from the previous task is used. The force transducer is fixed in a self-built wooden board which is placed on the table in front of the participants to prevent any movement of the transducer during the task (see Fig. 4). Experimental procedures, i.e., data acquisition, were programmed using a customized LabView 2015 (National Instruments, Austin, TA, USA) script. The task is to tap with the dominant index finger on the force transducer, which participants carry out in two different conditions: as consistently as possible at a self-selected pace, and tapping as fast as possible with disregard to consistency. Each trial lasts 15 s, with three trials in the first condition and two trials in the second condition. There is no visual feedback for the participants.

**Fig. 5:**
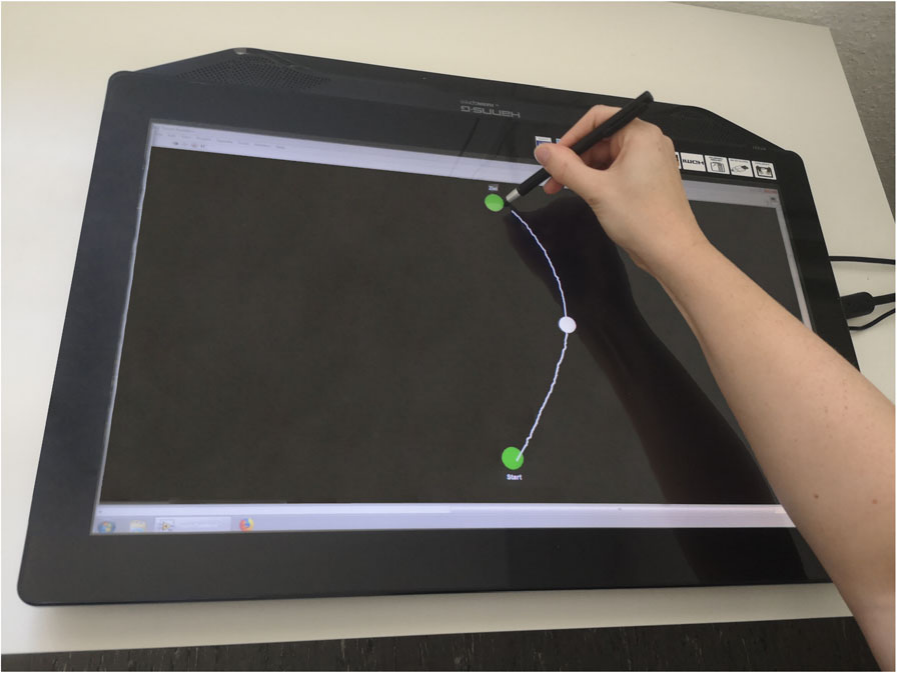
Set-up for fine motor task “connecting dots / tracing”

For the fine motor task (3) “connecting dots / tracing”, a touch monitor (Manufacturer: Hannstar Display Corp., 23.0 in., hardware resolution 1920 × 1080 pixels, Taipei City, Taiwan; Modell: HSG 1353) and a touch pen (WACOM Bamboo-Stylus Alpha CS-180, length 130 mm, diameter 9 mm, weight 12 g) are used. The monitor is placed horizontally on a table in front of the participants. The pen is held in the dominant hand (see Fig. 5). Experimental procedures, i.e., data acquisition and real time visual feedback, were programmed using a customized LabView 2015 (National Instruments, Austin, TA, USA) script. Participants have to connect dots on the touchscreen (black desktop background) via the touch pen by drawing a white line. There are two tasks: tracing a straight line and tracing a curved line. Two green dots (diameter 15 mm) are shown in the straight line setting, which are marked with 'Start' and 'Target', one above the other (see Fig. 5). Furthermore, there are two different distances (50 mm and 200 mm) between the start and target dots. In addition, a third white in-between-dot (diameter 12.5 mm) is presented half way between the start and target dots in each condition (horizontal distance 25% of that distance (12.5 mm or 50 mm) to the right) in the curved line setting, which the participants have to draw through (see Fig. 5). Overall, four conditions (straight line short, straight line long, curved line short, and curved line long) with seven trials are completed in randomized order.

#### Foot-eye coordination tests (pedal)

**Fig. 6:**
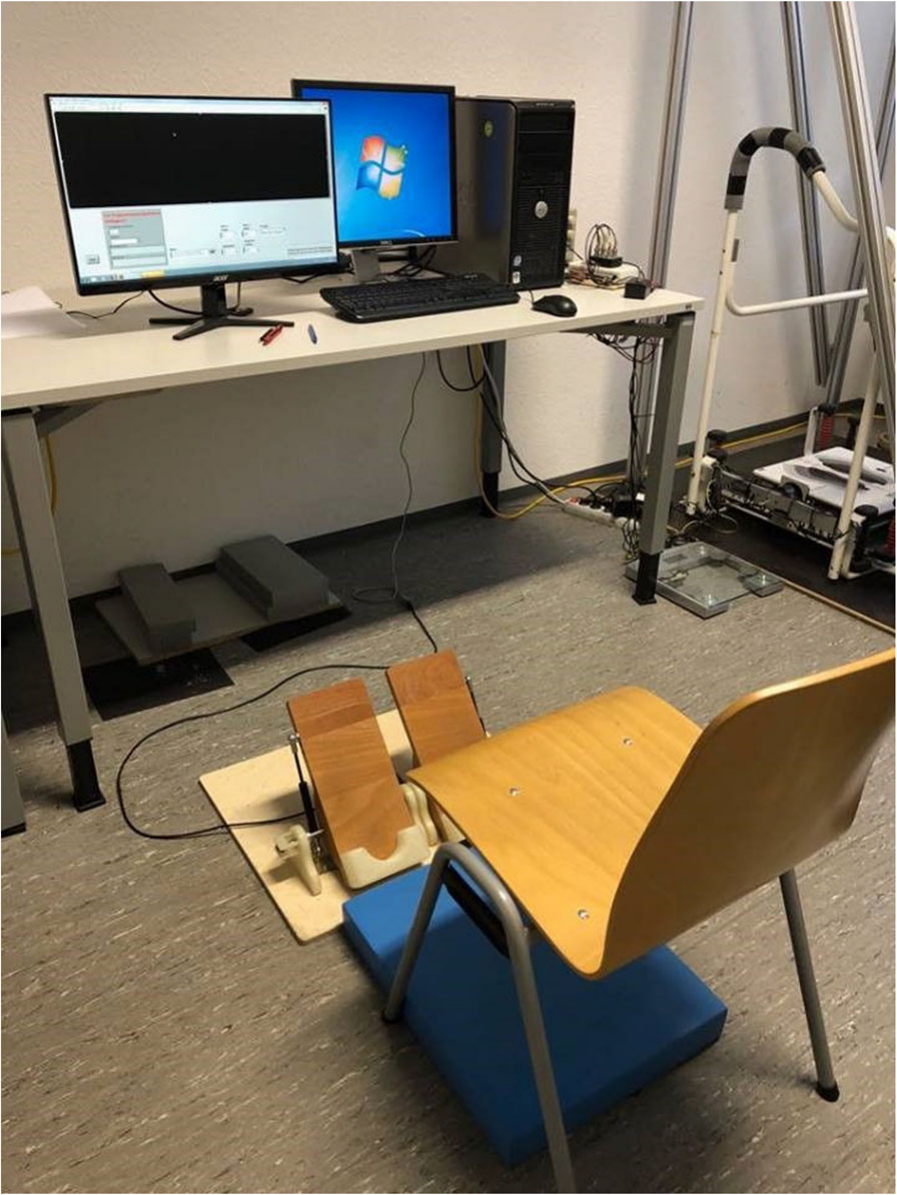
Set-up for foot-eye coordination

Foot-eye coordination (foot proprioception) is investigated using self-constructed pedals for the right and left feet (Fig. 6). The pedals are equipped with a gas spring (Febrotec, Nitrider® 0GS-N06AAA0050, Halver, Germany) and a linear potentiometer (Vishay Electronic GmbH, 249FGJS0XB25, 1kohm, Landshut, Germany). With a starting position of 45° dorsiflexion with respect to the horizontal floor, the pedals can be freely rotated within a range of ±20°. The analog data of the potentiometer is converted to digital signals using an AD card (Measurement Computing, USB-231, Bietigheim-Bissingen, Germany). A flat computer screen (24 in., 16:9) is positioned approximately 100 cm in front of the participant. The foot-eye coordination task is an accuracy task, in which participants are requested to reproduce given curves using their foot (right and left, separately). Visual feedback is provided on the screen using a routine in LabView 2015 (National Instruments Corp., Texas, USA). During plantar or dorsal flexion, the rotation of the pedals (pressing or depressing the pedal) generates live control of the line on the screen. Participants are instructed to reproduce the displayed curve as precisely as possible. The visual feedback begins as soon as the pedals are pressed.

For the foot-eye coordination tests, participants are instructed to sit comfortably, keeping their knees and hips at 90°, placing their feet on the pedals, and adjusting the distance between the chair and the pedals. Participants are instructed to reproduce given curves using their foot (right and left, separately) to operate the pedals. For each trial, ten sinusoidal curves at two different frequencies are displayed as a continuous graph. One test-trial per foot is performed. After the test-trial, three trials per foot are collected in a randomized sequence. Foot temperatures at the first metatarsal head (Met1) are measured before and after testing. Note that the three trials did not seem to be sufficient to promote long-lasting and relevant practice effects. Even with training sessions, a study of Teasdale et al. [83] exhibited no long-term retention in learning processes for MCI.

#### Functional status and physical performance

Functional status and physical performance is assessed using the Short Physical Performance Battery (SPPB), a standardized instrument which includes tests for (1) balance (legs closed / feet together, semi-tandem stand, tandem stand), (2) comfortable gait speed over four meters, and (3) chair raising test (five times sit-to-stand transfer) [84]. Each test is scored between 0 and 4, the total score ranges from 0 (low mobility/functionality) to 12 (full mobility / functionality).

Additionally, cardiovascular fitness is measured using the 2-min step test [85]. Therefore, the OA step in place as often as possible in 2 min. All steps with the right leg are scored.

Hand Grip Strength is assessed using the digital grip dynamometer (Grip D®, Takei scientific instruments, Niigata City, Japan). Three assessments are executed with the right and the left hand with a straightened elbow [86].

We also collect data for height, weight, and body fat using a stadiometer (seca213, seca Deutschland, Hamburg, Germany) and a bioimpedance scale (Tanita InnerScanV, Model BC-545 N, TANITA Corporation, Tokyo, Japan).

### Sensory measures

#### Visual acuity

The Freiburg Visual Acuity Test [87] with Landolt C is used to measure visual acuity. The participants are placed exactly 3 m from the screen and complete 18 trials. The measurement is carried out with vision aid to measure corrected vision.

#### Hearing

To measure corrected hearing ability, four lists of the Freiburg monosyllabic test (part of the Freiburg speech test [88]) are presented without background noise via headphones. Four different sound levels (35 dB, 47 dB, 24 dB, 53 dB) are used in the same order for all participants.

#### Vibration perception thresholds

**Fig. 7:**
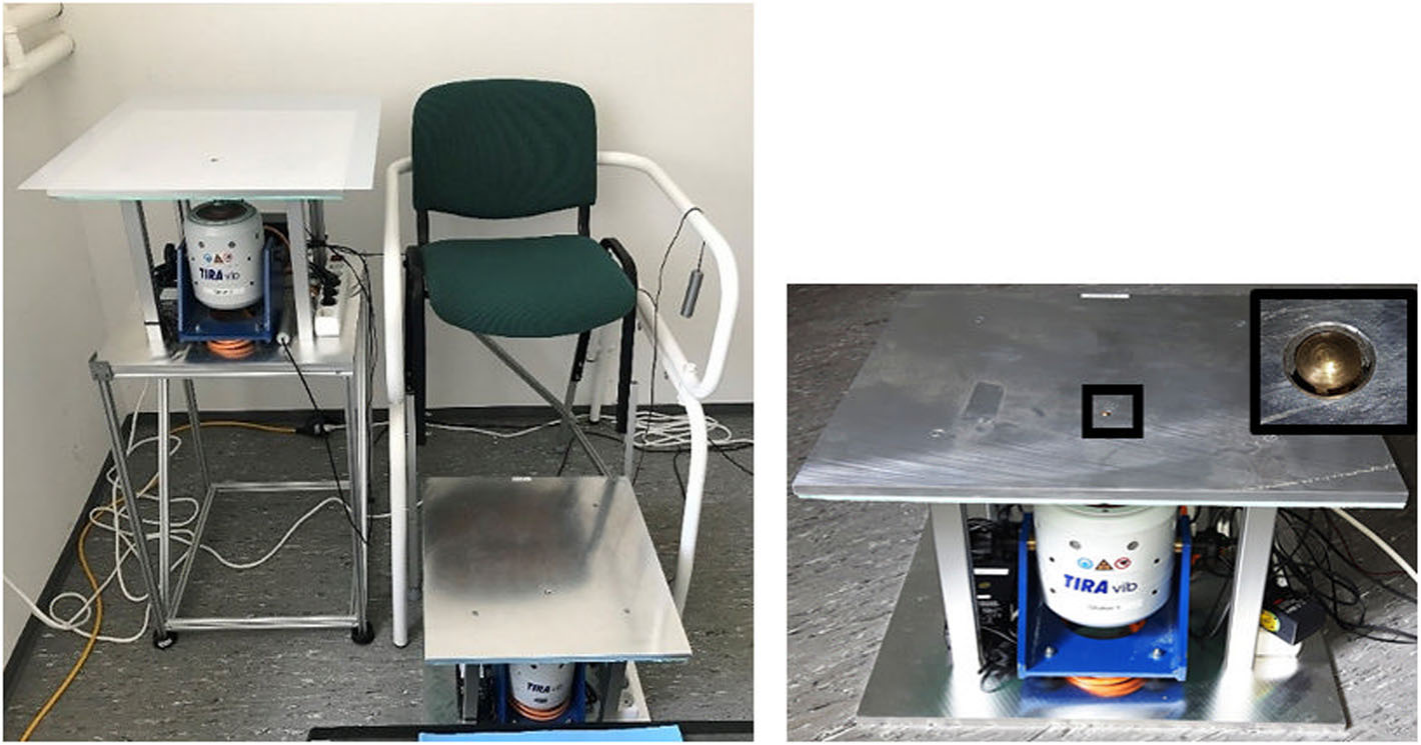
Left: Vibration perception threshold set-up for measuring hand and foot sensitivity. Right: Platform with tip of vibrating probe (black squares)

To assess skin sensitivity, vibration perception thresholds (VPTs) are measured using a Tira Vib vibration exciter (model TV51075, Schalkau, Germany) (Fig. 7), which presents good reliability [89]. Vibration from the exciter is applied to the foot location by a metal probe (rounded, 7.8 mm diameter) protruding through a hole (2 mm above surrounding surface level), according to [32, 90]. The surface of the vibration exciter is an aluminum platform equipped with heating elements to maintain the surface at a constant temperature (in this case 25 °C), to avoid skin temperature fluctuations. Vibration amplitude (in μm) is detected using an accelerometer (MMA2241KEG, NXP Semiconductors). The frequency of the vibrating contactor is set at 30 Hz and 200 Hz, which are known to be the optimal stimuli to elicit Meissner corpuscles and Vater–Pacini corpuscles, respectively [91]. The vertical force applied from the participants’ feet toward the probe is monitored via a force transducer and kept within a range of ± 0.5 N. Acoustic noise cancelling headphones (Bose® QuietComfort 25) are used to ensure that there is no distraction during the measurements.

##### Vibration perception threshold tests

First, sensitivity tests at the fingertip are performed. Participants are instructed to sit in a standardized manner but also comfortably, to be able to concentrate on detecting the vibration stimuli. The fingertips rest on top of the metal probe, without exerting additional pressure. Furthermore, participants wear acoustic noise cancelling headphones. Before starting the tests, test-trials are performed to define the value of the starting amplitude for the consecutive trials. The protocol for measuring VPTs is similar to a method of limits approach introduced by Mildren et al. [92].

In short, vibrations are introduced above the threshold, so that they can be clearly perceived by the participants (start amplitude defined according to test-trials). For each trial, a sequence of vibrations with different amplitudes (with randomized pauses in between) is applied and participants are asked to push a hand-held button as soon as they perceive each vibration stimulus. After pressing the button, the intensity of the previous, perceivable vibration stimulus is halved. When a vibration stimulus is not perceived, the next stimulus is delivered at half the intensity of the unperceived and the previously perceived stimuli. Then, four more stimuli are delivered to determine the final VPT. In total, three VPT-trials are collected at the fingertip of the index finger (at 30 Hz). Skin temperatures at the fingertip are measured before and after the three trials. After the hand sensitivity tests, the same protocol is performed to test foot sensitivity at the first Metatarsal head (at 30 Hz and 200 Hz).

### Questionnaire battery

Participants need to complete a questionnaire battery, which includes the following secondary outcome parameters: frailty, physical activity, social support, social activities, depression, comorbidities, health behavior, quality of life, and handedness. The self-administered questionnaire contains validated instruments and self-generated items which are shown in Table 4. Sociodemographic information includes age, sex, education, and employment.

**Table 4:** Outcome measures in the self-administered questionnaire

Outcome measure	Instrument/scale
**Physical activity**	
Physical activity	Modified Baecke Inventory (similar to [93]) PRISCUS-Physical Activity Questionnaire [94] Nürnberger-Alters-Inventar (NAI) [95]
**Social support, social activities**	
Social support	Social Support Questionnaire - short form [96]
Social activities	Florida Cognitive Activities Scale (modified [97])
**Health behavior**	
Objective health	List with diseases and use of medication (modified [98])
Comorbidities	Charlson Comorbidity Index [99]
Chronic medication	Individual medication regimen (name, dosage and frequency of intake for all prescribed medication)
Frailty	Frail Scale [100, 101] Tilburg Frailty Indicator [102, 103]
History of falls	Elderly Fall Screening Test (modified [104])
Falls efficacy	Fall Efficacy Scale [105]
Smoking behavior	Smoking Behavior Questionnaire [106]
**Quality of life and well-being**	
Quality of life	Satisfaction with Life Scale [107]
Depression	Geriatric Depression Scale [108]
Personality	Big Five Inventory [109, 110]
**Handedness**	
Handedness	Edinburgh Handedness Inventory [111]
Manual activities	Manual Activities Questionnaire [112]

### Data collection and management

Participant information will be recorded by a coded ID number. Hard copy forms will be stored in locked cabinets accessible only by project staffs. Electronic data will be stored on a secured computer that is password-protected. The databases will not contain subject identifiers and the data linking subject identifiers and the subject ID code will be stored separately.

Data quality will be promoted by double data entry and range checks for data values.

Only project staffs will have access to the final trial dataset.

### Data monitoring

A data monitoring committee, responsible for data monitoring, interim analyses, and auditing, will not be established, because no adverse events are to be expected. However, study participants will be under the surveillance of trained project staff who will intervene if a negative reaction is observed during the measurements.

### Sample size

The sample size calculation was based on the outcome cognitive decline. Based on literature [47, 51] small to moderate effect sizes are expected. Statistical power analysis using G*Power (Version 3.1.9.4, Franz Faul, University of Kiel, Germany) showed that 200 participants are required for analysis with α = .05 and power = .80. Expecting a 20% dropout rate during the study period, 240 participants will be included.

### Statistical analyses

A multiple regression model (with e. g. ordinary least squares technique) is used to detect several predictors or mediators of cognitive decline. To identify the most parsimonious model, and with it the final predictors, we analyze the corresponding coefficients of determination and consider the multiple comparisons problem providing a proper method to counteract it. Additionally, we propose an alternative classification taking hand of the k-nearest neighbors algorithm. Furthermore, we analyze changes in motor, sensory, electroencephalogical, and cognitive parameters over time in all three groups using mixed-effects models to explain the correlations in repeated measures in the same subject. Hazard ratios of progressing to dementia for participants with cognitive, motor, sensory, and neurophysiological decline are obtained in the classical way using the Cox semi-parametric proportional hazard model. Several variables are included as potential confounders, such as sex, age, education, comorbidities, psychological status, and social support. The most appropriate procedure for handling missing data will be selected after inspecting the amount and pattern of missing data.

### Expected results

We expect to find several motor, sensory, electroencephalogical, and cognitive prodromal markers for early detection of dementia and its pre-stages. Our assumptions are based on a current literature overview including international and national study results [18, 19, 23, 113].

### Trial registration

The trial was retrospectively registered at German Clinical Trials Register (DRKS) with registration number DRKS00013167 (https://www.drks.de/drks_web/navigate.do?navigationId=trial.HTML&TRIAL_ID=DRKS00013167; http://apps.who.int/trialsearch/Trial2.aspx?TrialID=DRKS00013167; Date of registration 11 April 2018).

## Conclusion

This study aims to detect possible motor, sensory, electroencephalogical, and cognitive predictors to develop a screening tool for dementia and its pre-stages in older adults, aged ≥80 years. Thus, affected individuals could receive optimal health care at an earlier stage to better-maintain their health resources. Nevertheless, some study limitation have to be mentioned. First, cognitive decline will be determined based on the results of cognitive instruments (MoCA and CERAD-Plus) and not based on imaging or cerebrospinal fluid measures [9]. Next, participation in the study is voluntary and the participants have to come to the labs by themselves. This may lead to an inadvertent recruitment of persons with higher cognitive or physical performance levels. Due to the funding period of 3 years and the different time points of study recruitment, it is not possible to observe cognitive decline of the participants over an extended period. Despite of the use of reliable and valid instruments to detect predictors for an early screening tool for cognitive decline, practice effects cannot be excluded completely. In spite of these limitations, a longitudinal design clearly outweighs a cross-sectional one. The present study is one of few studies [53, 54] investigating cognitive, motor, sensory, and neurophysiological markers in combination to develop a multi-dimensional instrument to predict cognitive decline or dementia.

## Data Availability

All participant information and data will be stored securely and identified by a coded ID number only to maintain participants’ confidentiality. Data can be obtained from the corresponding author upon reasonable request.

## Abbreviations

ADL: Activities of daily living
CERAD-Plus: Consortium to Establish a Registry for Alzheimer’s Disease
CHI: Cognitively healthy individuals
FLei: Fragebogen zur geistigen Leistungsfähigkeit
MCI: Mild cognitive impairment
MoCA: Montreal Cognitive Assessment
OA: Older adults
SCI: Subjective cognitive impairment
SENDA: Sensor-based systems for early detection of dementia
SST: Serial Sevens Test
VFT: Verbal Fluency Test
